# 

**Published:** 1889-06

**Authors:** 


### THE NATIONAL ASSOCIATION OF DENTAL EXAMINERS.

ORANGE, N. J., June 15th, 1889.

The next meeting of the National Association of Dental Examiners will be held in Saratoga, N. Y., Tuesday, August 5th, 9.30 A. M., and at other times during the week, between the sessions of the American Dental Association. It is important to have every State Board represented.

FRED. A. LEVY, D. D. S., *Sec.*

### To Readers and Correspondents.

Communications intended for publication, and exchange journals and papers, should be addressed to the Editor of The American Journal of Dental Science, No. 845 N. Eutaw St. Baltimore, Md. All letters relating to the business of the Journal, or containing cash remittances, to be directed to Messrs. Snowden & Cowman, No. 9 W. Fayette Street, Baltimore, Md. Articles for publication should be received by the Editor at least two weeks previous to the first of the month on which the number in which it is designed for them to appear, is issued.

☞ The American Journal of Dental Science will contain fifty pages of reading matter in each number, making a volume of about Six Hundred pages,

EXCLUSIVE OF ADVERTISEMENTS.

